# Risk factors for the development of *Clostridioides difficile* infection in patients colonized with toxigenic *Clostridioides difficile*

**DOI:** 10.1017/ice.2025.4

**Published:** 2025-06

**Authors:** Josh Clement, Gauri Barlingay, Sindhu Addepalli, Heejung Bang, Monica A. Donnelley, Stuart H. Cohen, Scott Crabtree

**Affiliations:** 1 Department of Pharmacy, University of California Davis Health, Sacramento, CA, USA; 2 Department of Pharmacy, Mount Sinai Hospital, New York, NY, USA; 3 Division of Infectious Disease, University of California Davis Medical Center, Sacramento, CA, USA; 4 Department of Internal Medicine, University of California Davis, Sacramento, CA, USA; 5 Division of Biostatistics, University of California Davis, Davis, CA, USA

## Abstract

**Objective:**

Asymptomatic patients colonized with toxigenic *Clostridioides difficile* are at risk of progressing to *C. difficile* infection (CDI), but risk factors associated with progression are poorly understood. The objectives of this study were to estimate the incidence and identify risk factors to progression of hospital-onset CDI (HO-CDI) among colonized patients.

**Methods:**

This was a nested case-control study at an academic medical center including adult patients colonized with toxigenic *C. difficile*, detected via polymerase chain reaction (PCR) on a rectal swab collected on admission from 2017 to 2020. Patients with prior CDI or symptoms on admission, neutropenia, prior rectal surgery, or hospitalization less than 24 hours were excluded. Colonized patients that developed HO-CDI were matched 1:3 to colonized patients who did not based on PCR test date. Bivariate and multivariable-adjusted Cox regression analyses were used to identify risk factors.

**Results:**

Of 2,150 colonized patients, 109 developed HO-CDI, with an incidence of 5.1%. After exclusions, 321 patients (69 with HO-CDI) were included, with an estimated incidence of 4.2%. Risk factors included cirrhosis (aHR 1.94), ICU admission (aHR 1.76), malignancy (aHR 1.88), and hospitalization within six months (aHR 1.6). Prior antibiotic exposure in the past three months (aHR 2.14) and receipt of at-risk antibiotics were also identified as potential risk factors (aHR 2.17).

**Conclusions:**

Progression to HO-CDI among colonized patients was not uncommon. This study highlights key risk factors associated with progression, underscoring the importance of enhanced monitoring and prevention efforts tailored to high-risk populations to mitigate HO-CDI.

## Introduction

*Clostridioides difficile* infection (CDI) is a leading cause of health care–associated infections, resulting in substantial morbidity and mortality worldwide.^1,2^ Asymptomatic individuals colonized with *C. difficile* do not display signs of CDI, but can act as reservoirs for transmission. Asymptomatic *C. difficile* colonization occurs in 3%–21% of adults in acute care hospitals.^3^ Rates are lower when excluding non-toxigenic strains, which are not associated with CDI progression. About 22% of individuals colonized with toxigenic strains progress to CDI (RR 5.86, 95% confidence interval (CI), 4.21–8.16), but incidence varies significantly.^4^ The risk factors that lead to progression from carrier state to acute infection are not well characterized, with limited studies to date specifically examining the population of individuals colonized with toxigenic *C. difficile*.^3,5–9^ Identifying factors associated with progression to CDI among patients colonized with toxigenic *C. difficile* is imperative given the role asymptomatic carriage plays. Studying this population can inform targeted interventions and identify prevention strategies to prevent CDI progression. At our institution, a universal rectal swab polymerase chain reaction (PCR) surveillance test is performed on all adult patients upon admission to determine toxigenic *C. difficile* colonization status. Colonization status is documented in the electronic medical record (EMR) and patients are placed on contact enteric precautions per institutional protocols. This practice, adopted from findings that demonstrated the utility of universal screening for mitigating *C. difficile* transmission, was implemented as part of our infection prevention strategies.^10^ Among a cohort of colonized patients, we attempted to study the incidence, characteristics, and risk factors for progression to CDI.

## Methods

### Study design, definitions & population

We conducted a nested case-control study of patients with positive *C. difficile* admission screening at the University of California Davis Medical Center between November 2017 and December 2020. Patients were identified through an EMR-generated report. Patients ≥ 18 years old with a positive *C. difficile* toxin PCR on admission were included. Patients were excluded if they had any documented history of CDI identified through EMR or external medical records, presented with diarrhea or other gastrointestinal symptoms consistent with community-onset CDI, or if their PCR screen was performed > 24 hours after admission. Patients with prior CDI were excluded to minimize confounding impact on future CDI risk and reduce misclassification of residual DNA from recent infection as colonization. Patients hospitalized < 24 hours, pregnant, with an absolute neutrophil count < 500, or with a history of rectal surgery were excluded as they do not routinely undergo screening at our institution. Exclusions were applied during initial testing and chart review. Patients who developed hospital-onset (HO-CDI) were selected in a 1:3 ratio to toxigenic *C. difficile* colonized patients who did not develop HO-CDI, matched by PCR test date to control for temporal changes, ensuring same-month admissions. The study was approved by the Institutional Review Board.

Asymptomatic *C. difficile* colonization was defined as a positive *C. difficile* rectal swab PCR without CDI symptoms. CDI was defined as 1) diarrhea (≥ three loose stools within 24 hours) without any other etiology and with a positive *C. difficile* toxin enzyme immunoassay (EIA); 2) pseudomembranous colitis on colonoscopy; or 3) histopathologic diagnosis. PCR-positive/toxin-negative samples were not routinely retested unless clinical suspicion for CDI remained high. HO-CDI was defined as CDI with symptoms appearing ≥ 72 hours after admission. Incidence was calculated using proportional retention rates (63% for cases, 77% for controls) and applied to the total screened population to estimate the retained cohort. HO-CDI incidence was calculated as the proportion of retained patients who developed HO-CDI. Detailed calculations are in the supplementary material (Appendix S1).

The primary outcome was HO-CDI incidence, and we aimed to identify risk factors associated with development of HO-CDI. Secondary outcomes included admission all-cause mortality, hospital length of stay (LOS), and discharge disposition.

### Clinical data collection

Data collected included baseline demographics, comorbidities, admission origin (home, transfer from an outside hospital, or admission from a skilled-nursing facility, long-term care, or rehabilitation center), recent hospitalization, surgical history, medication use (opioids, proton-pump inhibitors [PPI], immunosuppressants), antibiotics received within 90 days prior to admission and during hospitalization up to HO-CDI diagnosis, antibiotic class, hospital and ICU LOS, and antibiotics received during admission until HO-CDI diagnosis. Prior antibiotic use was identified through chart review within our healthcare system, including linked outpatient dispense reports and external records accessed via the Care Everywhere network. For non-HO-CDI, LOS reflects total hospitalization.

### Laboratory assays

Colonization status was detected via rectal/fecal swab tested for the *tcdB* gene using PCR (Xpert *C. difficile* test; Cepheid, Sunnyvale, CA). Testing for CDI was performed on unformed stool samples using a two-step algorithm; glutamate dehydrogenase (GDH) testing followed by confirmatory EIA (C. Diff Quik Chek Complete; TechLab, Blacksburg, VA), consistent with guideline-recommended algorithms by performing a highly sensitive test first, followed by a highly specific test.^11^

### Statistical analyses

Descriptive statistics characterized the study sample. Categorical variables were summarized by frequencies and percentages and compared using the χ^2^ test or Fischer’s exact test. Continuous variables were summarized by means and standard deviations (SD) and compared using logistic regression. To identify factors associated with HO-CDI, bivariate and multivariable Cox proportional hazard models were performed to estimate unadjusted and adjusted associations, respectively. To select factors independently associated with HO-CDI, covariates with a *P*-value < .10 in the bivariate analysis and/or factors identified by Poirier *et al.* were included in the multivariable Cox model.^8^ Individual antibiotic classes were excluded from regression models due to multicollinearity, multiplicity, and limitations in assessing antibiotic effects. At-risk antibiotics were defined as penicillins, cephalosporins, carbapenems, quinolones, macrolides, trimethoprim-sulfamethoxazole, or clindamycin, based on their known association with subsequent CDI and continuing prior work by Poirier *et al.* ^8^ The following risk factors were included in the model regardless of *P*-value, due to previously reported associations with CDI: age, opioid use, and PPI use.^8,12–14^ A time-dependent covariate for the time to first dose of an at-risk antibiotic was included in the Cox model. Unadjusted/crude hazard ratios (HRs) and adjusted hazard ratios (aHRs) with 95% CI were computed. CI and *P*-values were not adjusted for multiplicity. All analyses were conducted using SAS version 9.4 (SAS Institute Inc., Cary, NC) and BlueSky Statistics version 7.40 software (BlueSky Statistics LLC, Chicago, IL).

## Results

### Characteristics of Clostridioides difficile colonized patients

**Figure 1.**
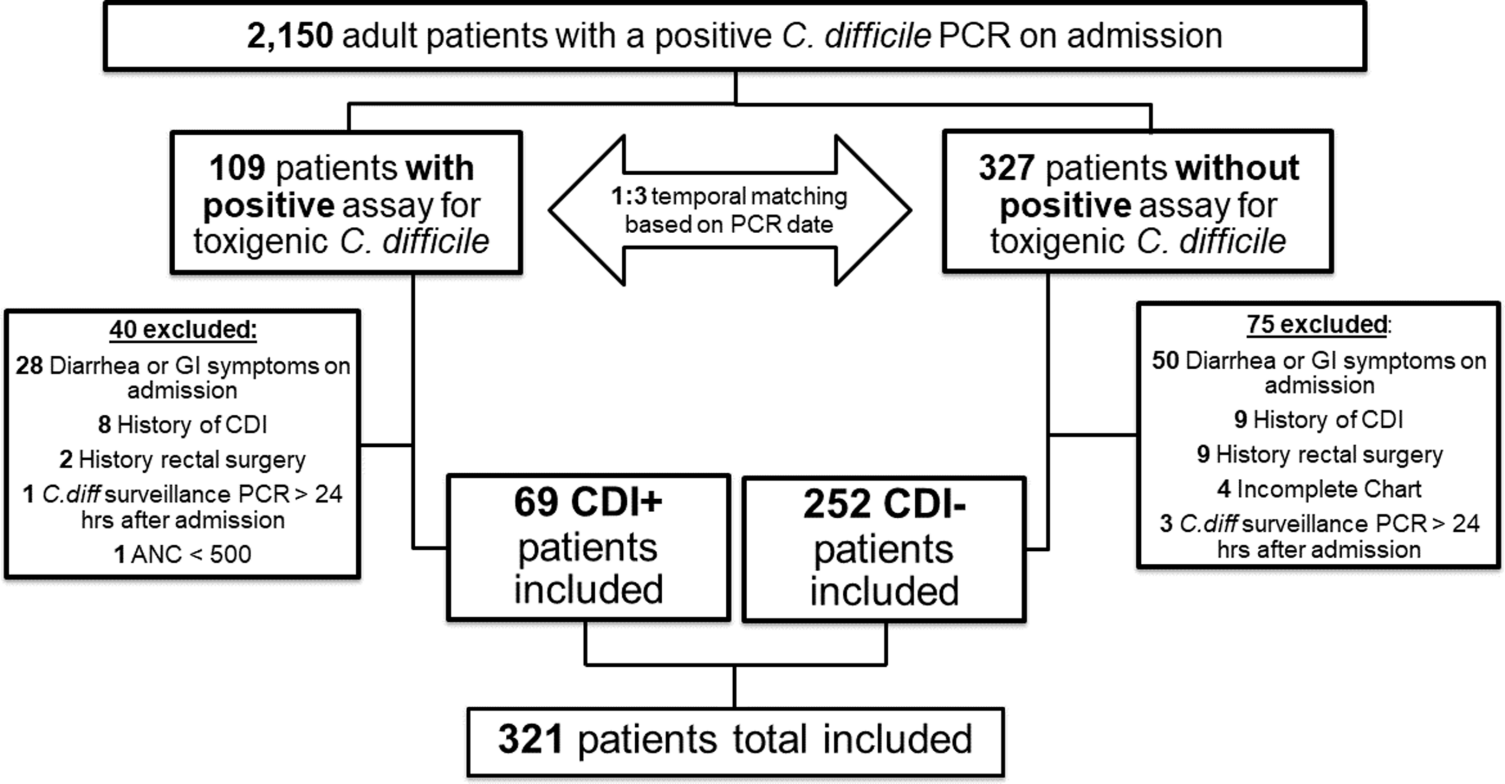
Study flow diagram. UC Davis Medical Center, November 2017 to December 2020. Abbreviations: PCR, polymerase chain reaction; *C. difficile*, *Clostridioides difficile*; CDI, *Clostridioides difficile* infection; GI, gastrointestinal; ANC, absolute neutrophil count.

During the study period, 57,468 patients were screened and 2,150 (3.7%) were colonized with toxigenic *C. difficile* via rectal PCR swabs. Among them, 109 patients were diagnosed with CDI based on symptoms and a positive EIA toxin assay. These cases were matched 1:3 to 327 EIA toxin-negative or untested patients by PCR test date. After exclusion, 321 patients comprised the final cohort (Figure 1). Baseline characteristics by CDI status are in Table 1. The mean age was 64 years (SD 16.1), 45% were female, and the average hospital LOS was 11.9 days (SD 20.7). Most patients (70.1%) were admitted from home, 37.1% to the ICU on admission, and 61.7% had prior hospitalization within six months. Additionally, 78.8% received antibiotics during admission.

**Table 1.** Clinical characteristics of patients colonized with *Clostridioides difficile*

Characteristic	Overall population, N = 321	No HO-CDI, n = 252	HO-CDI, n = 69	OR (95% CI)	*P* value^a^
Demographic information					
Age, mean (SD), years	64 (16.1)	63.4 (16.5)	66.3 (14.1)	1.01 (0.99–1.03)	.19
Age ≥ 65, No. (%)	166 (51.7)	123 (48.8)	43 (62.3)	1.73 (1.01–2.99)	.048
BMI, mean (SD), kg/m^2^	27.2 (8.2)	27.5 (8.8)	25.9 (5.6)	0.97 (0.94–1.01)	.14
BMI ≥ 30 kg/m^2^, No. (%)	98 (30.5)	83 (32.9)	15 (21.7)	0.57 (0.30–1.06)	.08
Female sex, No. (%)	144 (44.9)	111 (44)	33 (47.8)	1.16 (0.68–1.99)	.58
Hospital LOS Pre-CDI, mean (SD), days^a^	8.6 (12.9)	–	7.4 (11.7)	0.99 (0.96–1.02)	.40
ICU LOS Pre-CDI, mean (SD), days^b^	7.5 (14.7)	–	8.7 (13.1)	1.01 (0.98–1.03)	.34
Ethnicity, No. (%)					
Not Hispanic or Latino	272 (84.7)	213 (84.5)	59 (85.5)	*Ref*	
Caucasian	5 (1.6)	4 (1.6)	1 (1.5)	0.90 (0.10–8.23)	.93
Hispanic or Latino	40 (12.5)	31 (12.3)	9 (13)	1.05 (0.47–2.32)	.91
Declined to state	4 (1.2)	4 (1.6)	0 (0)	–	
Point of origin for admission, No. (%)					
Home	225 (70.1)	184 (73)	41 (59.4)	*Ref*	
Other healthcare institution^c^	96 (29.9)	68 (27)	28 (40.6)	1.85 (1.06–3.22)	.03
Hospitalization in the past 6 Months	198 (61.7)	137 (54.4)	61 (88.4)	6.40 (2.94–13.93)	< .001
ICU Admit on Admission	119 (37.1)	80 (31.7)	39 (56.5)	2.80 (1.62–4.82)	< .001
Comorbidities, No. (%)					
Hypertension	216 (67.3)	172 (68.3)	44 (63.8)	0.82 (0.47–1.43)	.48
Asthma	21 (6.5)	20 (7.9)	1 (1.4)	0.17 (0.02 –1.29)	.09
Congestive Heart Failure	90 (28)	68 (27)	22 (31.9)	1.27 (0.71–2.26)	.42
Cirrhosis	30 (9.3)	18 (7.1)	12 (17.4)	2.74 (1.25–6.00)	.01
CKD ≥ Stage III	112 (34.9)	85 (33.7)	27 (39.1)	1.26 (0.73–2.19)	.40
COPD	56 (17.4)	42 (16.7)	14 (20.3)	1.27 (0.65–2.5)	.48
Diabetes Mellitus	111 (34.6)	76 (30.2)	35 (50.7)	2.38 (1.38–4.1)	.002
ESRD on HD	49 (15.3)	36 (14.3)	13 (18.8)	1.39 (0.69–2.8)	.35
HIV	7 (2.2)	5 (2)	2 (2.9)	1.47 (0.28–7.77)	.65
Inflammatory Bowel Disease	10 (3.1)	6 (2.4)	4 (5.8)	2.52 (0.69–9.21)	.23
Malignancy	57 (17.8)	37 (14.7)	20 (29)	2.37 (1.27–4.44)	.01
Prior Gastrointestinal Surgery	69 (21.5)	52 (20.6)	17 (24.6)	1.26 (0.67–2.35)	.47
Stroke	41 (12.8)	30 (11.9)	11 (15.9)	1.40 (0.66–2.97)	.38
Medications, No. (%)					
Chronic Steroids	32 (10)	22 (8.7)	10 (14.5)	1.77 (0.80–3.94)	.16
Immunosuppressants	35 (10.9)	20 (7.9)	15 (21.7)	3.22 (1.55–6.7)	.001
Opioids	218 (67.9)	175 (69.4)	43 (62.3)	0.73 (0.42–1.27)	.26
Proton pump inhibitor^d^	186 (57.9)	138 (54.8)	48 (69.6)	1.89 (1.07–3.34)	.03
Systemic Antibiotics, No. (%)					
In the Past 3 Months	163 (50.9)	110 (43.8)	53 (76.8)	4.25 (2.30–7.83)	< .001
During Admission (Prior to CDI)	253 (78.8)	194 (77.0)	59 (85.5)	1.76 (0.85–3.67)	.13
Composite^e^	285 (88.8)	220 (87.3)	65 (94.2)	2.36 (0.81–6.93)	.12
Time to first dose of at-risk antibiotic, mean (SD), days	0.97 (1.85)	0.98 (1.75)	0.97 (2.12)	1.00 (0.85–1.17)	.97
No. of antibiotic classes at risk for CDI, No. (%)^f^					
0	44 (13.7)	40 (15.9)	4 (5.8)	*Ref*	
1	94 (29.3)	83 (32.9)	11 (15.9)	1.33 (0.40–4.42)	.65
2	81 (25.2)	69 (27.4)	12 (17.4)	1.74 (0.53–5.75)	.36
3 or more	102 (31.8)	60 (23.8)	42 (60.9)	7.00 (2.33–21.04)	< .001

Abbreviations: HO-CDI, Hospital onset *Clostridioides difficile* infection; CI, confidence interval; CKD, chronic kidney disease; COPD, chronic obstructive pulmonary disease; ESRD, end-stage renal disease; HD, hemodialysis; HR, hazard ratio; HIV, human immunodeficiency virus; ICU, intensive care unit; LOS, length of stay; No, number; PCN, penicillin; Ref, reference category; SD, standard deviation.

a *P*-values are not adjusted for multiplicity.

b Compared to total mean LOS in No HO-CDI group since CDI did not occur.

c Defined as a transfer from an outside hospital, or admission from a skilled-nursing facility, long-term care, or rehabilitation center.

d Includes if patient was taking prior to admission or during admission.

e Includes antibiotics received in the past 3 months prior to admission and during admission.

f Includes all penicillins, cephalosporins, carbapenems, quinolones, macrolides, trimethoprim-sulfamethoxazole, and clindamycin.

### Patient outcomes

Among 2,150 total colonized patients, 69 developed HO-CDI following screening and exclusion, resulting in an estimated incidence of 4.2% (Table 2). The median time to diagnosis of HO-CDI was 7.4 (SD 11.7) days and all-cause mortality during admission was higher in HO-CDI patients (15.9% vs 6.3%; *P* = .011). Hospital LOS was longer in the HO-CDI group (22.7 vs 8.9 days; *P <* .001), and they were less often discharged home (42.0% vs 64.3%; *P <* .001).

### Risk factors associated with CDI

Bivariate analysis showed HO-CDI patients were more often ≥ 65 years (62.3% vs 48.8%; *P* = .048), admitted from healthcare institutions (40.6% vs 27%; *P* = .03), admitted to ICUs at baseline (56.5% vs 31.7%; *P* < .001), and hospitalized in the past six months (88.4% vs 54.4%; *P <* .001). They were also more likely to have cirrhosis (17.4% vs 7.1%, *P* = .01), diabetes (50.7% vs 30.2%, *P* = .002), malignancy (29% vs 14.7%, *P* = .01), and to have received the following medications: immunosuppressant prior to admission (21.7% vs 7.9%, *P* = .001), PPIs (69.6% vs 54.8%, *P* = .03), antibiotics three months prior to admission (76.8% vs 43.8%, *P* < .001), and ≥ three at-risk antibiotic classes during admission (60.9% vs 23.8%, *P* < .001). Receipt of antibiotics during hospital stay (85.5% vs 77.0%, *P =* .13), LOS prior to HO-CDI, age, and inflammatory bowel disease were not associated with an increased risk of HO-CDI.

The multivariable Cox model (Table 3) identified the following as significantly associated with progression to HO-CDI: cirrhosis (aHR, 1.94; 95% CI, 1.01–3.76; *P* = .049), hospitalization in the past six months (aHR, 2.72; 95% CI, 1.19–2.22; *P* = .026), ICU admission (aHR, 1.76; 95% CI, 1.07–2.89; *P* = .027), malignancy (aHR, 1.88; 95% CI, 1.06–3.33; *P* = .031), increasing number of at-risk antibiotic classes (aHR per each additional class, 2.17; 95% CI, 1.09–4.29; *P* = .026), and prior antibiotics (aHR, 2.14; 95% CI, 1.14–4.01; *P* = .017). Notably, opioid use was associated with a significantly lower risk of progression to HO-CDI (aHR, 0.34; 95% CI, 0.19–0.58; *P* < .0001). Age per year increase, immunosuppressants, and PPIs were not statistically significant.

**Table 2.** Outcomes of patients colonized with hospital-onset *Clostridioides difficile*

Outcome	Overall population, N = 321	No HO-CDI, n = 252	HO-CDI, n = 69	*P* value^a^
All-cause mortality, No. (%)	27 (8.4)	16 (6.3)	11 (15.9)	.011
LOS Post-CDI, mean (SD), days^b^	10.3 (18.4)	–	15.3 (30)	.009
ICU LOS Post-CDI, mean (SD), days^b^	6.2 (13.5)	–	4.7 (8.1)	.02
Total LOS, mean (SD), days	11.9 (20.7)	8.9 (13.3)	22.7 (34.7)	<.001
Total ICU LOS, mean (SD), days	8 (14)	6.9 (15.5)	9.9 (10.6)	.002
Discharge disposition, No. (%)				<.001
Home	191 (59.5)	162 (64.3)	29 (42)	
Skilled Nursing Facility	82 (25.5)	55 (21.8)	27 (39.1)	
Expired	25 (7.8)	15 (6)	10 (14.5)	
Hospice^c^	6 (1.9)	3 (1.2)	3 (4.3)	
Other	12 (3.7)	12 (4.8)	0 (0)	
Against Medical Advice	5 (1.6)	5 (2)	0 (0)	

Abbreviations: HO-CDI, Hospital onset *Clostridioides difficile* infection; CI, confidence interval; ICU, intensive care unit; LOS, length of stay; No, number; SD, standard deviation.

a *P*-values are not adjusted for multiplicity.

b Compared to total mean LOS in No HO-CDI group.

c Two of these patients subsequently expired prior to leaving the hospital.

**Table 3.** Multivariable-adjusted cox proportional hazards model of risk factors associated with hospital-onset *Clostridioides difficile* infection among colonized patients

	Outcome of HO-CDI		
Risk factor^a^	aHR	95% CI	*P* Value
Age per 1 year increase^b^	1.01	0.98–1.02	.850
Cirrhosis	1.94	1.01–3.76	.049
Hospitalization in the past 6 months	2.72	1.19–6.22	.018
ICU Admission	1.76	1.07–2.89	.027
Immunosuppressant Use	1.81	.96–3.39	.066
Malignancy	1.88	1.06–3.33	.031
Receipt of an at-risk antibiotic	2.17	1.09–4.29	.026
Opioid	0.34	0.19–0.58	<.0001
Prior antibiotic exposure^c^	2.14	1.14–4.01	.017
Proton pump inhibitor	1.27	0.72–2.23	.404

Abbreviations: aHR, adjusted hazard ratio; HO-CDI, Hospital onset *Clostridioides difficile* infection; CI, confidence interval; ICU, intensive care unit.

a All risk factors on admission or prior to CDI onset.

b Per one year increase.

c In the past 3 months.

All other factors are binary, *ie*, presence vs others. Antibiotic was coded as time-varying covariate/indicator during follow-up time; ie, 0 before receipt and 1 after receipt.

Detailed antibiotic data is available (Supplement Fig S1.) but should be cautiously interpreted due to potential confounding and collinearity with other risk factors and multiplicity. For example, composite metronidazole use was associated with increased HO-CDI risk on bivariate analysis, but on further review was almost always used in conjunction with other high-risk antibiotics like ceftriaxone or fluoroquinolones.

## Discussion

Our study identified several CDI risk factors among colonized patients, confirming associations from prior studies.^6–9^ Prior antibiotic exposure and the number of at-risk antibiotic classes used were major risk factors. Modifying antibiotic selection, particularly by reducing high-risk antibiotic use, may reduce CDI incidence.^15–17^ This is a modifiable risk that can be an effective intervention for stewardship programs to target colonized patients. Notably, 78.8% of colonized patients received antibiotics during hospitalization, a rate higher than the reported ∼50% in hospitalized patients.^18^ This underscores the need for stewardship efforts to minimize unnecessary antibiotic use and reduce progression to HO-CDI in this high-risk population.

Bivariate analysis found that patients ≥ 65 years were more likely to progress to CDI. However, similar to recent findings, multivariate analysis did not show increasing age as a CDI risk factor, despite it being a common predictor in non-screened populations.^2,6^ This adds to growing evidence that age may be associated with CDI due to higher colonization risk, rather than an increased risk of progression to CDI post-colonization.^8,19,20^

The association between *C. difficile* colonization and CDI in the ICU has shown mixed results in studies.^9,21–23^ Our analysis found that baseline ICU admission was independently associated with progression to HO-CDI in colonized individuals, even after controlling for other variables. ICU patients pose a challenge to stewardship programs due to acuity of illness, often necessitating the use of broad-spectrum antibiotics and hesitancy to de-escalate.^24,25^ Nevertheless, our findings indicate that such efforts are crucial in this population due to their elevated risk of CDI.

As commonly observed, immunosuppressant use and malignancy were associated with progression to HO-CDI.^26–28^ These populations are vulnerable due to altered immune responses, prophylactic antibiotics, frequent hospitalizations, and microbiome disruption.^26–28^ Notably, malignancy remained a significant risk factor after adjustment. Given the high incidence and poor outcomes, further studies are needed to determine whether certain populations with toxigenic *C. difficile* colonization, such as those with malignancy, might benefit from primary CDI prophylaxis. The role of oral vancomycin prophylaxis remains controversial due to its potential to disrupt the microbiome, warranting careful consideration of its risks and benefits, though fidaxomicin has shown potential for primary prophylaxis in non-screened, high-risk stem cell transplant patients.^29^

Studies have linked PPI use to CDI, potentially due to reduced gastric acid suppression leading to loss of protective effects or alternations in gut microbiota.^30–32^ However, the American College of Gastroenterology advises against discontinuing PPIs in CDI patients if indicated, given the relatively lower risk versus other factors.^33^ We found that PPI use (prior to or during admission) was not independently associated with progression to CDI. Despite this, PPI stewardship may still represent a valuable intervention, as studies suggest PPIs are unnecessary in over half of CDI cases though further validation is needed.^34^

Prior studies have linked recent hospitalization to both increased *C. difficile* colonization and infection at admission.^35,36^ In our study, hospitalization within the past six months was independently associated with progression to CDI in colonized patients. Patients with frequent healthcare-associated exposures may have elevated CDI risk due to unaccounted comorbidities, and the context of colonization exposure may serve as a surrogate for severity of illness.

Our study identified cirrhosis as a risk factor for CDI, consistent with prior findings in colonized patients.^8^ Cirrhosis may increase CDI risk due to use of antibiotics for treatment or prophylaxis of spontaneous bacterial peritonitis, PPI use, frequent hospitalizations, and gut microbiome alterations.^37^ Notably, cirrhosis remained a significant risk factor after adjusting for these confounders. However, the impact of lactulose was not assessed and may have influenced the observed association.

The relationship between opioid use and CDI progression in colonized patients has yielded mixed results. In our study, opioid use was associated with a reduced risk of CDI, conflicting with Poirier *et al.* findings of increased risk.^8^ The reason for this difference, despite similar study designs, is unclear but raises questions about the association’s veracity. Patients with opioid use disorder, particularly those with opioid-associated constipation or withdrawal treatment, may be less likely to undergo testing, potentially leading to an underestimation of CDI incidence.

This study builds on previous work in the toxigenic *C. difficile* colonized population. Lin *et al* assessed 86 medical ward patients, 14 of whom developed CDI, identifying diabetes, PPI use, and exposure to multiple antibiotic classes as risk factors.^6^ Poirier *et al* assessed 513 carriers admitted to their hospital, of which 39 developed CDI, and found associations with hospital LOS, cirrhosis, probiotics, opioids, and the number of at-risk antibiotic classes.^8^ The strengths of our study, which builds upon this literature, includes its larger sample size - the largest cohort to date, a broad sample population, use of a highly specific diagnostic testing algorithm, use of antibiotic as a time-varying covariate, and evaluation of hospital LOS as a risk factor rather than an outcome. Poirier *et al’s* diagnostic approach, which involved only a one-step PCR-based test or a compatible clinical illness in colonized individuals (15% of CDI cases), may have overestimated CDI incidence (7.6% vs 4.2%).^8^ Prior studies suggest that the clinical course of PCR-positive, toxin-negative cases often mirrors that of non-CDI diarrhea patients.^38,39^

There are important limitations to this study. Case-control studies are well-suited for exploring associations but may not provide unbiased estimates of population-based parameters and outcomes. The retrospective design also introduces well known potential biases. Misclassification is inherent to CDI diagnosis, given the imperfect sensitivity and specificity of all testing algorithms, and in our study some toxin-negative patients may have had undiagnosed CDI. At our institution, GHD+/toxin- results are reported as negative, so some GDH+/toxin- patients may have been included in the control group if they met inclusion criteria. This dichotomous reporting of CDI test results as “positive” or “negative” limits the ability to evaluate the clinical significance of GDH+/toxin- results, potentially leading to misclassification. No specific steps were taken to mitigate this, a limitation reflecting the inherent challenges of CDI diagnosis in practice. Nevertheless, both empiric treatment for CDI in colonized patients and CDI testing solely due to colonization are rare at our institution, and CDI testing due to colonization is strongly discouraged through clinical decision support. Furthermore, upon review no controls received CDI treatment during admission, supporting low clinical suspicion for CDI. We did not assess risk adjustment scores (eg, Charlson Comorbidity or Elixhauser Comorbidity Index), which could better estimate the impact of comorbidities. We were unable to evaluate antimicrobial exposure by number of doses or duration, limiting our ability to assess these effects on progression to CDI. Prior antibiotic use was identified through chart review, including linked outpatient pharmacy dispense reports and external records from outside institutions. However, this approach may not have captured all external antibiotic use, particularly for patients without linked records, potentially underestimating exposure. Patients with neutropenia or prior rectal surgery, populations potentially at increased risk for HO-CDI, were also excluded from screening limiting applicability to these groups. Lastly, generalizability may be limited, as this study was conducted at a center performing universal *C. difficile* screening on all admitted patients, a practice that may not be feasible at other institutions

This study provides important implications for hospitals developing infection control policies aimed at reducing HO-CDI in patients colonized with toxigenic strains. The identified risk factors, including patient-specific comorbidities and modifiable medication-related risks, can be easily identified and inform the development of a scoring system. However, until such system is prospectively validated, it is difficult to ascertain how best stewardship programs can intervene on these identified risks. Furthermore, many institutions have developed CDI scoring systems that perform poorly when externally validated.^40^ These points highlight the challenge of CDI prevention and underscore the need for institutions to locally validate their own patient-specific risk factors.

## Conclusion

Progression to HO-CDI occurred in 4.2% of toxigenic *C. difficile* colonized patients at our institution. This study highlights several factors associated with progression to CDI among colonized patients that stewardship programs can potentially target to decrease the risk of progression to HO-CDI. Given the significant risk of HO-CDI in these individuals, particularly those identified with risk factors, further investigation into the utilization of tailored stewardship interventions or primary prophylaxis is warranted.

## Supporting information

Clement et al. supplementary material Clement et al. supplementary material

## Supplementary material

The supplementary material for this article can be found at https://doi.org/10.1017/ice.2025.4
