# Supplementary material for: Risk factors for the development of Clostridioides difficile infection in patients colonized with toxigenic Clostridioides difficile
Source: Infect Control Hosp Epidemiol. 2025 Feb 24;46(6):597–603. doi: 10.1017/ice.2025.4 (PMC12169943; doi:10.1017/ice.2025.4)
Supplement: Clement et al. supplementary material [file S0899823X25000042sup001.docx]

**SUPPLEMENTARY MATERIALS**

**Title:** Risk Factors for the Development of Clostridioides difficile Infection in Patients Colonized with toxigenic Clostridioides difficile

**Authors:** Josh Clement,^1,5^ Gauri Barlingay,^2^ Sindhu Addepalli,^3^ Heejung Bang,^4^ Monica A. Donnelley,^1^ Stuart H. Cohen^2^, Scott Crabtree^2^

**Affiliations:**

1. Department of Pharmacy, University of California Davis Health, Sacramento, CA
2. Division of Infectious Disease, University of California Davis Medical Center, Sacramento, CA
3. Department of Internal Medicine, University of California Davis, Sacramento, CA
4. Division of Biostatistics, University of California Davis, Davis, CA
5. Department of Pharmacy, Mount Sinai Hospital, New York, NY

**S1.** Retention rates calculation

1. Retention rates (included/total screened):
   - Cases retained: $\frac{\text{69}}{\text{109}}$ = 63%
   - Controls retained: $\frac{\text{252}}{\text{327}}$= 77%
2. Application of retention rates to total cohort
   - Total controls initially identified prior to match: 2041
   - Retained controls: 2041 x 77% = 1572
3. Total retained patients: 69 (cases) + 1572 (controls) = 1641
4. Incidence calculation:
   - $\frac{\text{69}}{\text{1641}}$ x 100 = 4.2%


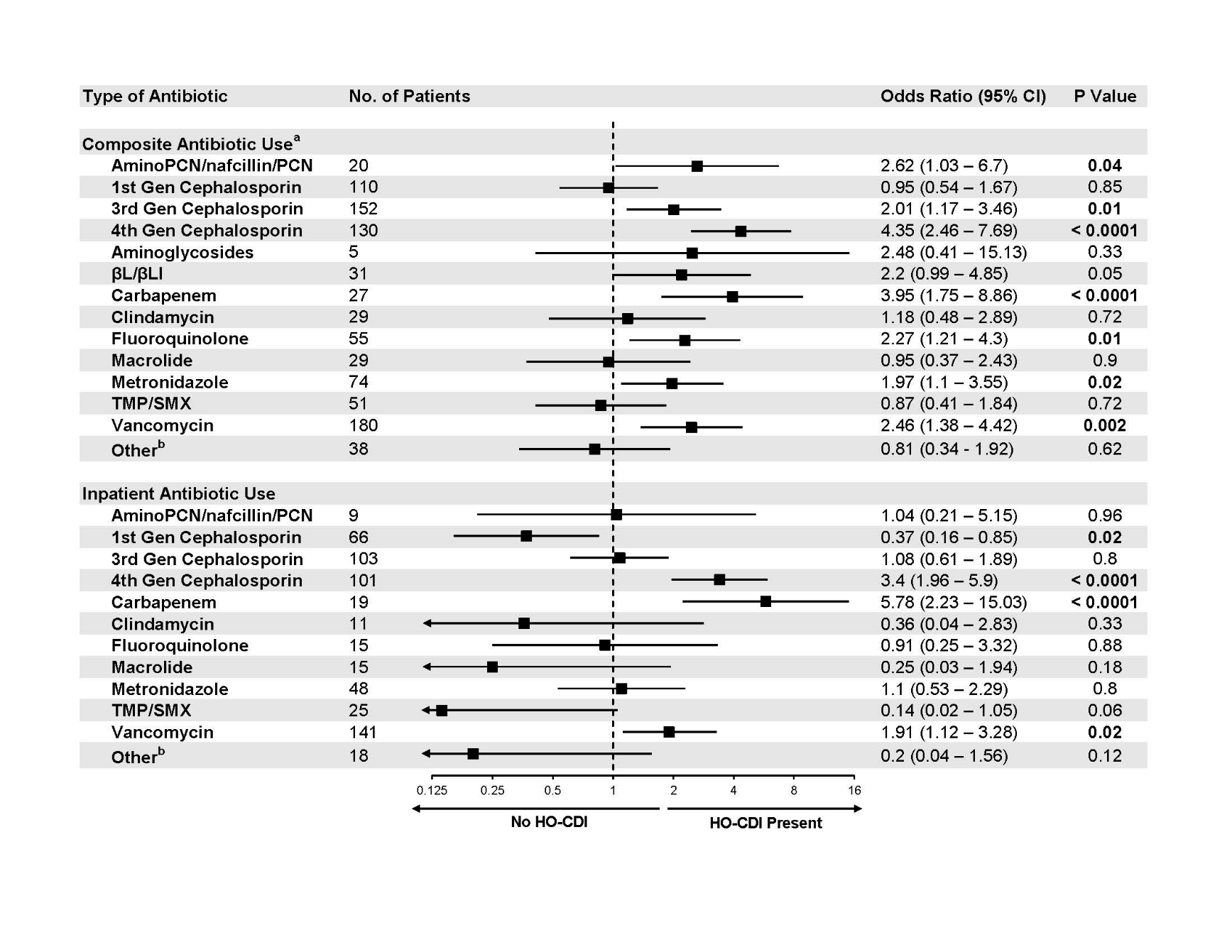
**Figure S1.** Forest plot of unadjusted ORs for 95% confidence intervals for *C. difficile* infection associated with different antibiotic classes by composite^a^ use and by inpatient admission use only.

**Abbreviations**: βL/βLI, beta-lactam/beta-lactamase inhibitor; CI, confidence interval; HO-CDI, hospital onset-*Clostridioides difficile* infection; PCN, penicillin; TMP/SMX, trimethoprim-sulfamethoxazole.

^a^Includes antibiotics received in the past 3 months prior to admission and during admission.

^b^Includes doxycycline, aztreonam, nitrofurantoin.

*P*-values and CIs were not adjusted for multiplicity. If adjusted using the Bonferroni method, the *P*-value threshold would be 0.0019 (0.05/26).
